# Phase-separated Ca and Mg-based nanoparticles in SiO$_{2}$ glass investigated by molecular dynamics simulations

**DOI:** 10.1038/s41598-022-16139-w

**Published:** 2022-07-13

**Authors:** Jorel Fourmont, Wilfried Blanc, Dominique Guichaoua, Stéphane Chaussedent

**Affiliations:** 1 Univ Angers, LPHIA, SFR MATRIX, 49000 Angers, France; 2 UMR 7010, CNRS, Institut de Physique de Nice (INPHYNI), Université Côte d’Azur, 06108 Nice, France

**Keywords:** Materials for optics, Nanoscale materials, Theory and computation

## Abstract

The development of new applications based on glass doped with nanoparticles is growing in interest during the last years. The structure and properties of Ca-based silicate nanoparticles formed in situ in a silica matrix through a phase separation mechanism were investigated by using Molecular Dynamics simulations and compared to nanoparticles formed from MgO-codoping. We showed that such nanoparticles have non-spherical shape, are amorphous and inhomogeneously distributed in the host glass. In this modeled structure, a release of non-bridging oxygen atoms, due to a depolymerization phenomenon of the nanoparticles’ silica network, was observed. Besides, we demonstrated that nanoparticles’ composition is size-dependent. Compared to Mg-silicate nanoparticles, Ca-based nanoparticles are larger, less concentrated in Ca, and we observed a steeper concentration change during the phase separation process. Those differences are related to the diffusion coefficients of Ca and Mg. This numerical analysis informs on the alkaline-earth nanoparticles’ properties within a host silica glass, which can be a relevant guide for the development of new materials for applications such as nanoparticle-doped optical fibers.

## Introduction

Recent advances in the development of doped glasses with nanoparticles (NPs), like glass-ceramics, phase-separated glasses or metallic-nanoparticles doped glasses, highlight an improvement of the material’s properties (optical, mechanical, thermal...) in many fields^1–4^. Specifically, material’s performance and development are driven by the engineering ability to control the NPs’ chemical composition, shape, and size. For example, a novel strategy consists of improving the luminescent properties of rare-earth ions in optical fibers made of phase-separated nanoparticles^5^. In this case, controlling the chemical composition, size and shape of NPs is essential because the luminescence properties of rare-earth ions are related to their local environment^6^, and light scattering should be minimized. On the contrary, new optical fiber sensors take advantage of the light scattering^7,8^. However, such NPs may be amorphous, and their small size makes their experimental characterization difficult. Consequently, Molecular Dynamics (MD) simulations turn out to be a helpful alternative to obtain information on the structural properties at the nanoscale. In a previous work, from MD simulations of rare-earth doped NPs formed in a Mg-silicate matrix^9^ we have shown an over-concentration of the rare-earth ions within the NPs and a reduction of the clustering effect in comparison with a simple rare-earth doped silica glass. A size-dependent composition of these NPs was also observed, in agreement with the experimental measurements of Blanc et al.^10^. It was also demonstrated that such rare-earth doped NPs can be formed using different divalent metals oxides, and especially with CaO^11,12^. Very recently, engineering of Rayleigh scattering was reported in Ca-based nanoparticles-doped optical fibers^4^. Therefore, it appears interesting to study the influence of the metal oxide on the structural properties of the NPs. In this context, MD simulations were performed using the modified version of the Pedone potential refined by Bidault et al.^9^ to reproduce a phase separation mechanism. By this way, Ca-based nanoparticles were formed in situ in a silica matrix glass of 0.10CaO–0.90SiO$_{2}$ chemical composition and elaborated through the classical melt/quench procedure. From the modeled structure, Ca-rich and Si-poor microphases were identified and defined as Ca-silicate nanoparticles. Afterwards, the structural and compositional properties of these NPs, as well as the local environment encountered by Ca$^{2+}$ cation were analyzed in detail. Finally, the NPs’ structural properties and the Ca$^{2+}$ behavior in the host matrix were compared with those encountered in Mg-based nanoparticles within a previously modeled 0.10MgO–0.90 SiO$_{2}$ glass structure^9^.

## Computational methods

### Force-field description

Historically, numerous interatomic potentials have been designed to investigate the structural and dynamical properties of amorphous silica and silicates systems. In a previous paper, Afify et al.^13^ showed the good accuracy of the Pedone potential to reproduce the structural and mechanical properties of silica glass using classical Molecular Dynamics (MD). The original version of the Pedone potential^14^ is a sum of three terms: a long-range Coulomb term, a short-range Morse function and a repulsive contribution of the form $C_{ij}/r^{12}$, in order to model the interaction at high temperature and high pressure:

1 $$\begin{aligned} V(r_{ij}) = \frac{q_{i}q_{j}}{r_{ij}} + D_{ij}\Big [\Big (1-e^{-a_{ij}(r_{ij}-r_{0})}\Big )^{2}-1\Big ] + \frac{C_{ij}}{{r_{ij}}^{12}} \end{aligned}$$

where $D_{ij}$, $C_{ij}$, $a_{ij}$ and $r_{0}$ are adjustable parameters, $r_{ij}$ is the interatomic distance of the i-j pairs and $q_{i}, q_{j}$ are the partial charges of ions i, j. The long-range electrostatic interactions are evaluated by means of the Wolf method^15^, whose scheme consist of a spherical truncation, charge-neutralization, shifting, and pairwise 1/r summation. The cutoff distance is set at 7.5 Å and a damping parameter of 0.30 Å is used, whereas the Morse interactions are truncated at 5.5 Å. To reproduce the phase separation phenomenon, it is essential to consider the different ionicity of the metal-oxygen bonds^16^ of the binary system. Bidault et al.^17^ refined the Pedone potential into a variable charge model: the cations charge is assumed to be constant, while each oxygen charge of the O-O repulsive interaction dynamically reacts with its own cationic environment. To this purpose, the charge of each oxygen atom is calculated as a negative weighted sum of the surrounding cationic charges, as following:

2 $$\begin{aligned} q_{o} = \sum _{\begin{array}{c} cations\\ r< r_c \end{array}} {f(r) \cdot q_{cation}}/{\sum _{\begin{array}{c} oxygens\\ r < r_c \end{array}} f(r)} \end{aligned}$$

Within a sphere of radius $r_{\hbox {c}}$ and centered on an oxygen atom, all positive charges are multiplied by a weighting function f(r) decreasing with r, where r is the distance between a cation and this central oxygen atom. Then, all oxygen atoms within the same radius $r_{\hbox {c}}$ are counted and weighted by the same function f(r), where r is the distance between an oxygen atom and the central oxygen. The weighting function f(r) is empirical and takes the form of a polynomial of order 3:

3 $$\begin{aligned} f(r) = \frac{1}{2}\Big [1+\cos \Big (\frac{\pi r}{r_{c}}\Big )\Big ] \approx 2\Big (\frac{r}{r_c}\Big )^{3}-3\Big (\frac{r}{r_c}\Big )^{2}+1 \end{aligned}$$

Finally, to ensure the electroneutrality of the system, all oxygen charges are scaled in such a way that the average value of the oxygen charge is equal to the average charge imposed by electroneutrality. The cutoff radius r$_{\hbox {c}}$ of the weighting function is empirically fixed at 7.5 Å. A complete description of the procedure can be found in a previous study on the MgO–SiO$_{2}$ binary mixture^17^. Following the protocol applied by Pedone et al.^14^, the Ca–O interatomic potential parameters have been fitted using the free energy minimization method^18^ implemented in the GULP code^19^ and performed on the crystalline structure of CaO lime available on MINCRYST crystallographic database^20^. New parameters for the Ca–O interaction are reported in Table 1, allowing thereby to reproduce accurately the nearest neighbor distances for Si and Ca, density $\rho$ and values of bulk modulus B for CaO lime^21^, calcio-olivine (Ca$_{2}$SiO$_{4}$)^22,23^, wollastonite (CaSiO$_{3}$)^24,25^ and pseudowollastonite^26,27^, which are reported in Table 2. Total structure factor F(Q) for CaSiO$_{3}$ glass and normalized total structure factor S(Q) for 3CaO–4SiO$_{2}$ glass were calculated and compared with experimental neutron diffraction data^28,29^ in the next section.

**Table 1:** Potential parameters for MD modelling of xCaO–(1-x)SiO$_{2}$ structures.

	$\hbox {D}_{ij}$ (eV)	$\hbox {a}_{ij}$ (Å$^{-1}$)	$\hbox {r}_{0}$ (Å)	$\hbox {C}_{ij}$ (eV Å$^{12}$)
Si$^{2.4}$–O$^{-1.2}$	*0.340554*	*2.006700*	*2.100000*	*1.0*
Ca$^{1.5}$–O$^{-1.5}$	**0.033747**	**1.646077**	**3.303187**	**0.0**
Mg$^{1.33}$–O$^{-1.33}$	*0.209290*	*1.376871*	*2.733041*	*0.0*
O$^{\hbox {q}}$–O$^{\hbox {q'}}$	*0.042395*	*1.379316*	*3.618701*	*22.0*

Ca–O interaction parameters are represented in bold text. Mg potential parameters (italic text) are also reported from previous study^9,17^. The non-coulombic interactions were zero for all cation-cation interactions. Values given in exponent of ion type are partial charges. q and q’: each oxygen partial charge depends on its own cationic environment.

**Table 2:** Comparison between experimental (plain text) and modeled (bold text) calcium silicate crystal structures: CaO^20,21^, Ca$_{2}$SiO$_{4}$^22,23^, CaSiO$_{3}$^24,25^ and pseudowollastonite^26,27^.

Structural parameter	CaO lime	Ca$_{2}$SiO$_{4}$ calcioolivine	CaSiO$_{3}$ wollastonite	CaSiO$_{3}$ pseudo-wollastonite
a (Å)	4.81	5.07	7.94	6.85
	**4.83**	**5.08**	**7.91**	*6.83*
Relative error (%)	*0.42*	*0.20*	− *0.38*	*0.29*
b (Å)	4.81	11.21	7.32	11.89
	**4.83**	**11.22**	**7.09**	**11.86**
Relative error (%)	*0.42*	− *0.09*	*3.18*	*0.25*
c (Å)	4.81	6.75	7.07	6.75
	**4.83**	**6.76**	**7.01**	**6.76**
Relative error (%)	*0.42*	− *0.15*	*0.85*	− *0.15*
$\rho$ (g cm$^{-3}$)	3.35	2.98	2.91	2.89
	**3.30**	**2.97**	**2.95**	**2.92**
Relative error (%)	*1.49*	*0.34*	− *1.37*	− *1.04*
r$_{\hbox {Si}{-}\hbox {O}}$ (Å)	–	1.61	1.62	1.62
	–	**1.60**	**1.60**	**1.61**
Relative error (%)	–	*0.62*	*1.23*	*0.62*
r$_{\hbox {Ca}{-}\hbox {O}}$ (Å)	2.40	2.38	2.40	2.53
	**2.40**	**2.39**	**2.41**	**2.55**
Relative error (%)	*0.00*	− *0.42*	− *0.41*	− *0.79*
B (GPa)	111	100	93	86
	**114**	**105**	**82**	**90**
Relative error (%)	− *2.70*	− *5.00*	*11.83*	− *4.65*

Relative error are indicated in italic text.

### Simulation setup

Glass modelling is done under isothermal-isobaric ensemble (NPT) using a Langevin thermostat (damping parameter = 100 fs) and a Berendsen barostat (damping parameter = 1000 fs) to control the thermodynamic quantities of the system. Equations of motion are integrated by the Verlet-Velocity algorithm^30,31^ with a timestep of 1 fs. Ca- and Mg-silicate glasses are prepared following the same melt-quench numerical protocol as the one applied previously^9^: starting from a silica glass, Ca (or Mg) and O atoms are placed randomly in the silica matrix to obtain the desired chemical composition. To make the initial silica glass model, we start from a crystalline configuration ($\alpha$-cristobalite). Then, we fix the size of the simulation box to match a density of 2.203, which is the current experimental density of silica glass. The simulation box is a 50.9245 Å edge cube containing 8748 atoms: 2916 Silicon and 5832 Oxygen. This structure is heated to 4000 K in order to ensure a homogeneous mixing of the system and a complete decorrelation from the original structure. Then, the liquid is cooled down in gradually and continuously reducing the temperature from 4000 to 300 K with a quench rate of 5$\times$10$^{11}$ K s$^{-1}$. Such a quenching rate is by several orders of magnitude faster than those applied in laboratory, but it is a common value for Molecular Dynamics glass simulations since it makes it possible to reproduce the expected experimental vitreous structure^32^. Then, the system is replicated 4 times along x and z directions, and Ca (or Mg) and O atoms are placed randomly in the silica matrix to obtain a system with a molar composition of 0.10MO–0.90SiO$_{2}$ (5184 M = Ca or Mg for a total of 150336 atoms) and a volume of approximately 200$\times$50$\times$200 Å$^{3}$, where periodic boundaries conditions (PBC) in the three orthogonal directions are applied. The system is melted again at 4000 K during 0.1 ns and quenched at 5$\times$10$^{11}$ K s$^{-1}$ from 4000 K to 2400 K. Above this temperature, only one single-phase solution exists. Afterwards, stages of 6.4 ns each are successively performed by step of 100 K, from 2400 K to 1900 K, a temperature range wherein the phase separation or metastable immiscibility occurs^33,34^. Under the glass transition temperature T$_{\hbox {g}}$, the system is practically frozen (for this quench rate, T$_{\hbox {g}}$ = 2240 K for 0.10CaO–0.90SiO$_{2}$ glass, against 2210 K for a 0.10MgO–0.90SiO$_{2}$ glass). Finally, the mixture is quenched at 5$\times$10$^{11}$ K s$^{-1}$ from 1900 K to 300 K. All results presented here are derived from this single modeled structure at 300 K and atmospheric pressure. Results from a previous study^9^ concerning 0.10MgO–0.90SiO$_{2}$ glass structure are used for comparison in the latest part of this paper. MD simulations were conducted using a modified version of LAMMPS program^35^ (LAMMPS file definition and modified files from the corresponding author are available on reasonable request) and OVITO^36^ was chosen as visualization software.

### Structural and dynamical analysis

#### Total neutron structure factor

**Figure 1:**
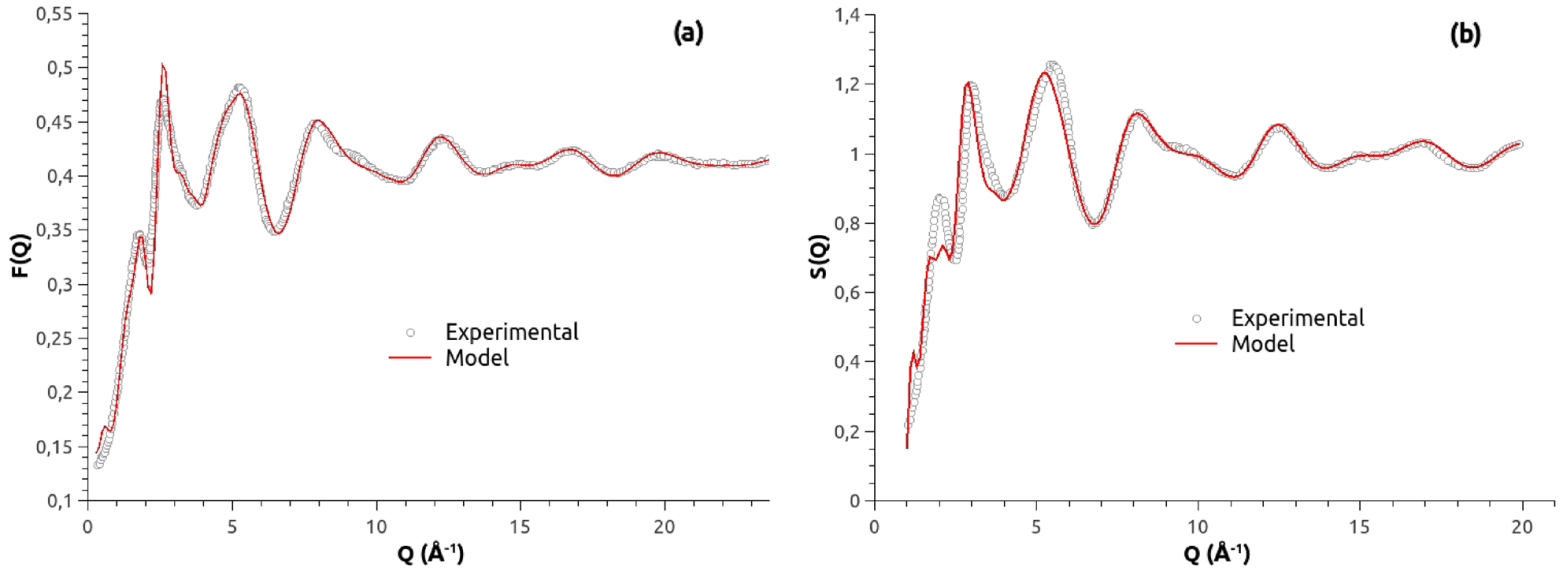
Comparison between the experimental structure factors (empty dot) derived from neutron diffraction and those calculated from the MD model (red lines) for (**a**) CaSiO$_{3}$ glass and (**b**) 3CaO–4SiO$_{2}$ glass.

The experimental neutron diffraction data have been compared to the data obtained from the two glasses structures modeled with the new potential parameters: CaSiO$_{3}$^29^ and 3CaO–4SiO$_{2}$^28^. Following the notation proposed by Keen^37^, the normalized total-scattering neutron structure factor S(Q) can be written in the Faber-Ziman formalism:

4 $$\begin{aligned} S(Q) = 1+\frac{F(Q)}{(\sum _{i=1}^{n} c_{i}\bar{b_{i}})^{2}} \end{aligned}$$

where ${c_{i}}$ and ${b_{i}}$ are the atomic concentration and the coherent scattering length for species i, respectively, and *Q* is the magnitude of the scattering vector. The coherent scattering lengths for Si, Ca and O species coincide with the values used by Cormier et al.^29^. *F(Q)*, the total-scattering neutron factor, is defined as:

5 $$\begin{aligned} F(Q) = \sum _{i,j}c_{i}c_{j}\bar{b_{i}}\bar{b_{j}}[F_{ij}(Q)-1] \end{aligned}$$

and ${F_{ij}(Q)}$ is related to the partial radial distribution functions, ${g_{ij}(r)}$, as follows:

6 $$\begin{aligned} F_{ij}(Q) = 1+\rho _{0}\int _{0}^{\infty }4\pi r^{2}[g_{ij}(r)-1]\frac{sin(Qr)}{Qr}dr \end{aligned}$$

with $\rho _{0} = N/V$ as the atomic number density. The corresponding neutron structure factors for the two simulated glasses, given in Fig. 1, agree with the experimental neutron structure factors and this confirms the relevance of the Ca–O parameters chosen in this work.

#### Q$^{{n}}$ distribution and oxygen speciation

**Figure 2:**
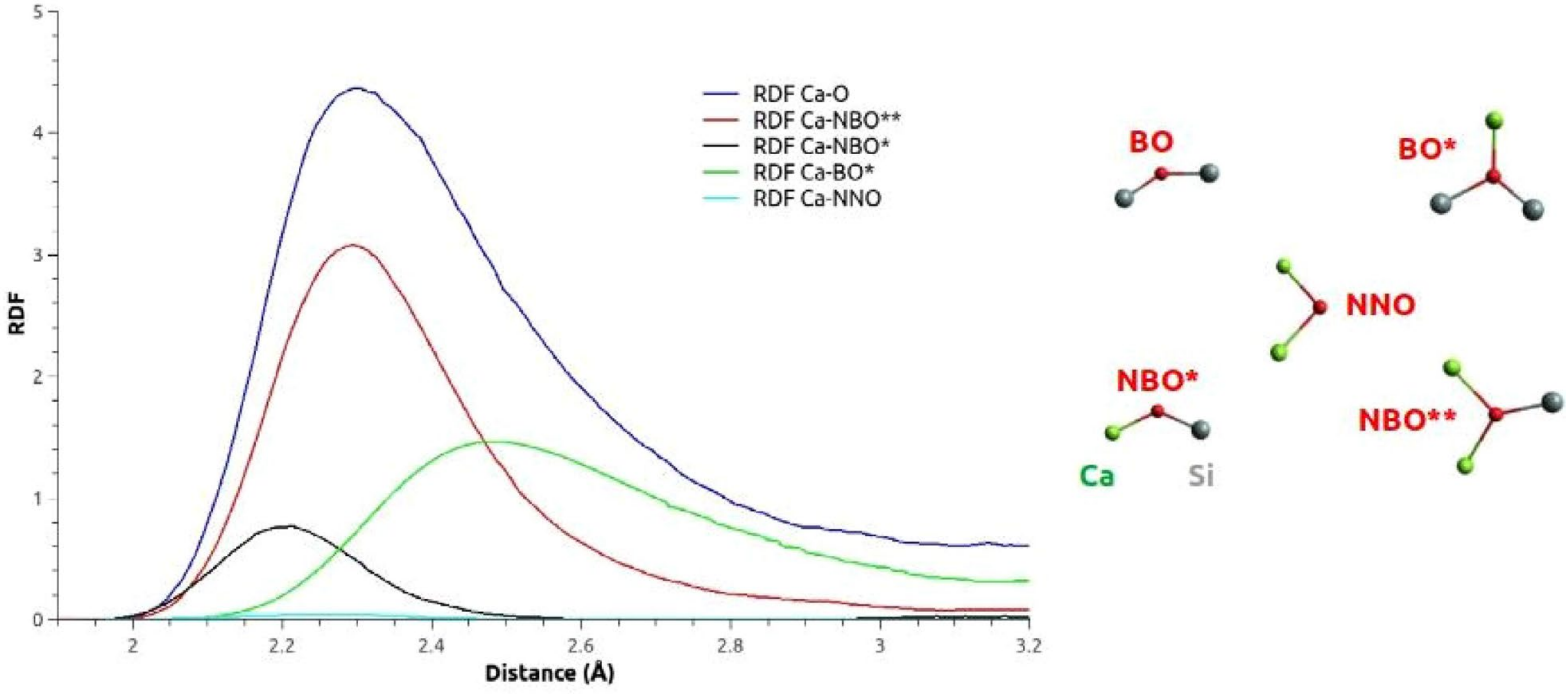
On the left side, different oxygen species contributions to the Ca–O RDF in the 0.10CaO–0.90SiO$_{2}$ glass. On the right side, schematic representation of the different oxygen species.

There are many models describing glass formation process and its aspects regarding nucleation and spinodal decomposition^38–43^. Generally, the local environment of silicon in silica glasses can be characterized by five Q$^{\hbox {n}}$ silicon species where n ranges 0-4 and corresponds to the number of bridging oxygen (BO) atoms per silicon tetrahedron. According to Hudon & Baker’s study^16^, the addition of modifier alkali or alkaline-earth oxide (in our case, Ca$^{2+}$ or Mg$^{2+}$), in lower quantities than the glass former, breaks oxygen bonds to form non-bridging oxygen (NBO), resulting in conversion of Q$^{\hbox {n}}$ species to Q$^{\hbox {n-1}}$ species and consequently, decreasing the connectivity of the network (i.e., depolymerization) in silicate melts. In a previous study^9^, it was showed that the Q$^{\hbox {n}}$ distribution is dependent on the MgO concentration because of the formation of NBO atoms with the addition of MgO. The same trend should be verified for CaO oxide. A complementary approach to study the cation local environment is to implement the terminology developed by Afify & Mountjoy^44^ to define the different oxygen species. As indicated below on the right side of Fig. 2: a BO bounded to one Ca atom is called BO*; a NBO bounded to one Ca atom is denoted NBO*, whereas a NBO bounded to two Ca atoms is denoted NBO**; finally, a Non-Network Oxygen (NNO) is an oxygen atom not bounded to any Si, but only to Ca atoms. The left side of Fig. 2 shows the Ca–O RDF and its contributions due to the previously defined oxygen species in the 0.10CaO–0.90SiO$_{2}$ glass model. The same discrimination has been previously applied for the 0.10MgO–0.90SiO$_{2}$ glass model^9^. We remark that the different linkages provided to the oxygen by its local environment induce very different first maximum positions of the contributions to the global CaO RDF, as it is the case in the 0.10MgO–0.90SiO$_{2}$ glass structure.

#### Self-diffusion coefficient

Using MD, the atomic transport coefficients can be determined from the long-time evolution of the mean-square displacement (MSD)^45^, as:

7 $$\begin{aligned} MSD \equiv <|r_{i}(t)-r_{i}(t_{0})|^{2}> = \frac{1}{N} \sum _{i=1}^{N}|r_{i}(t)-r_{i}(t_{0})|^{2} \end{aligned}$$

where ${r_{i}(t)}$ indicates the atom position. At long enough times, MSD exhibits a linear relationship with time and self-diffusion coefficient *D* can be extracted from the linear region of the mean-square displacement curve thanks to the Einstein relation, which is:

8 $$\begin{aligned} D = \lim _{t\rightarrow \infty }\frac{<|r_{i}(t)-r_{i}(t_{0})|^{2}>}{6t} \end{aligned}$$

The MSDs of Ca and Mg cations in 0.10MO–0.90SiO$_{2}$ glasses (M = Ca or Mg) were calculated for a duration of 1 ns to evaluate the self-diffusion coefficients and are discussed in the last part of the paper. Diffusivities provide an insight on the collective behavior of each modifier cation during the phase transition process, leading to the formation of nanoparticles in the studied silicate glasses.

## Results and discussion

### Nanoparticle’s description

**Figure 3:**
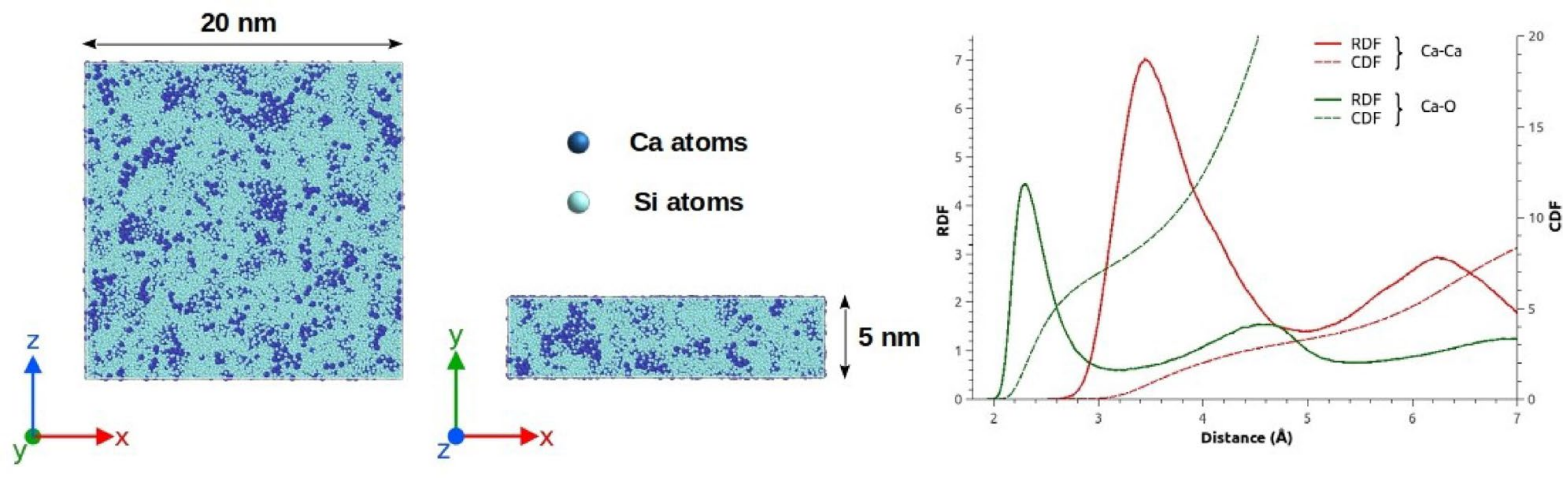
Visualizations of the 0.10CaO–0.90SiO$_{2}$ glass model (oxygen atoms are not represented for the sake of clarity), on the left side. RDF and CDF of the pair Ca–O and Ca–Ca in the 0.10CaO–0.90SiO$_{2}$ glass model, on the right side.

Snapshots of the phase separation in the 0.10CaO–0.90SiO$_{2}$ glass structure modeled by MD are displayed in the left side of Fig. 3. Two different region types are observed: Si-rich Ca-poor phases on one hand (represented in light blue) and Ca-rich Si-poor phases on the other hand (represented in dark blue). At a first sight, the Ca-rich Si-poor phases, defined as nanoparticles thereafter, seem to have non-spherical shape, to be amorphous and inhomogeneously distributed in the glass. The right side of Fig. 3 displays the Radial Distribution Functions (RDF) of the Ca–O and Ca–Ca pairs, as well as their associated Cumulative Distribution Functions (CDF) in the 0.10CaO–0.90SiO$_{2}$ glass model. Considering the Ca–Ca RDF, one can observe that the first peak is largely broadened around a mean distance of 3.5 Å, denoting that there is no crystalline arrangement in the CaO rich phases. On one hand, the Ca–Ca first peak position and the Ca–O mean distance ($\approx$ 2.34 Å) are closed to the experimental values obtained from EXAFS ($\approx$ 2.36 Å)^46^ and neutron diffraction ($\approx$ 2.37 Å)^47^ experiments in a CaSiO$_{3}$ glass. On the other hand, the adaptive Pedone potential also reports the short-range order structure as well as with a fixed charge model for the 0.10CaO–0.90SiO$_{2}$ glass^48^. The advantage of this adaptive potential is that it also takes account for phase separation process, while a fixed charge potential fails. To highlight this ability, snapshots of 0.10MgO–0.90SiO$_{2}$ glasses, modelled in the same conditions, without and with the adaptative part of the Pedone potential can be found in Supplementary Information S1.

**Figure 4:**
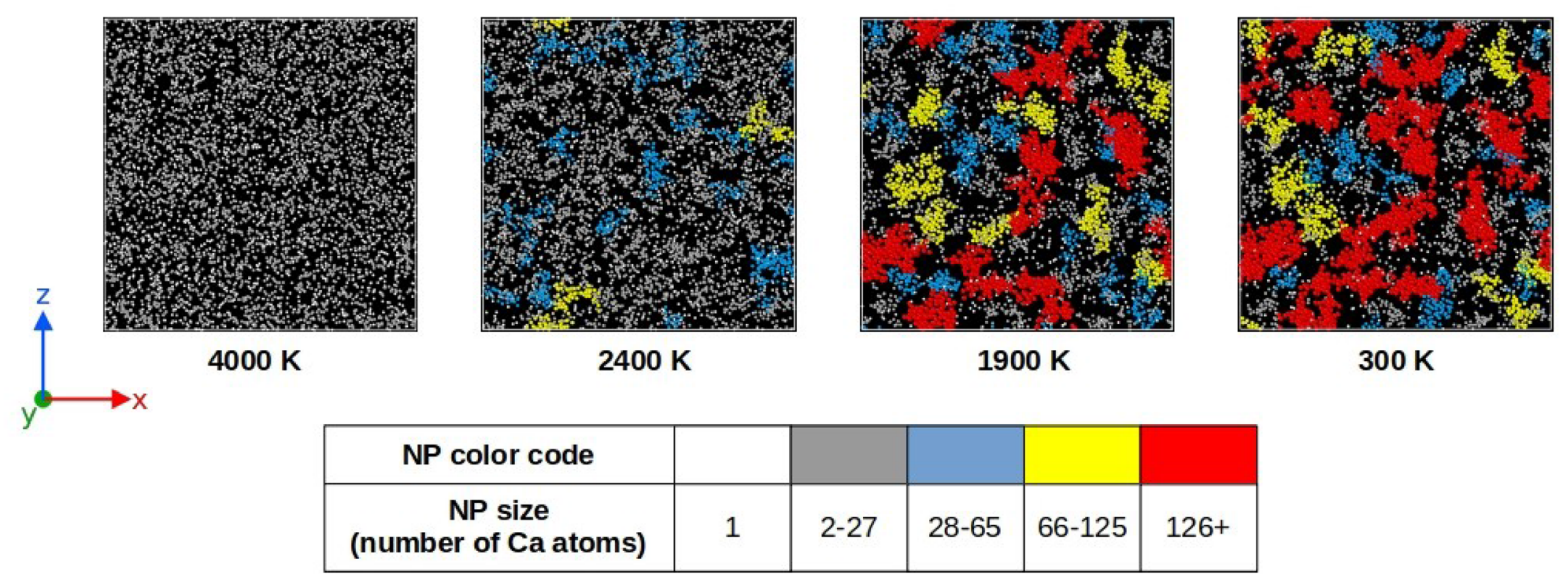
Snapshots showing the formation of nanoparticles in the 0.10CaO–0.90SiO$_{2}$ glass model during the glass formation process. Only Ca atoms are represented with a color code related to the size of the nanoparticle (Si and O atoms are not represented for the sake of clarity).

Figure 4 displays snapshots of NPs’ formation in the 0.10CaO–0.90SiO$_{2}$ glass structure where only Ca atoms are represented with a color code related to the size of the Ca-rich phase in which they are located. Two Ca atoms are considered belonging to the same NP if they are bounded to the same oxygen atom. Consequently, a Ca atom belongs to a NP if it is located at a distance shorter than a cut-off of 3.05 Å from an O atom of the NP. This cut-off distance is defined by the first minimum of the Ca–O RDF (see Fig. 3). Starting from a homogeneous melt, we observe that the formation of NPs occurs at around 2400 K, within a temperature interval where the phase separation allows the growing of the NPs. Below this temperature, the system seems to be almost frozen and only small variations of the NPs size are observed. The final structure exhibits NPs’ sizes ranging from a few Ca atoms to more than one hundred: about 7 % of Ca atoms are isolated in silica matrix whereas about 70 % of Ca atoms are involved into NPs composed of 28 Ca atoms and more.

The NPs’ composition as a function of NPs’ size in the 0.10CaO–0.90SiO$_{2}$ glass model is reported in Table 3. To determine the NPs’ composition, one considers that a Si atom belongs to a NP if it is distant from an oxygen atom of the NP by a length shorter than the first minimum of the Si-O RDF. In such a way, it is possible to determine the number of Ca and Si atoms of each NP. We notice that the CaO concentration of a NP increases with its size, ranging from 15.7 ± 2.6 mol% in the smallest NPs to 24.3 ± 1.0 mol% for the largest ones. In a recent paper, Ballato et al.^49^ have studied and designed a CaO-derived optical fiber, where a concentration of 25.9 mol% was found for the CaO high content microphase, that is in agreement with our MD results. In addition, the size-dependent composition of MgO-based NPs formed in situ in a silica matrix was already observed, by means of MD simulations^9^ or experiments^10^.

**Table 3:** Nanoparticle’s composition according to their size in the 0.10CaO–0.90SiO$_{2}$ glass model.

NP size (number of Ca atoms)	% [CaO]	% [SiO$_{2}$]	Standard deviation (%)
2–27	15.7	84.3	$\pm 2.6$
28–65	22.6	77.4	$\pm 1.4$
66–125	23.2	76.8	$\pm 1.6$
126+	24.3	75.7	$\pm 1.0$

### Structure of calcium environment

The distribution of Q$^{\hbox {n}}$ species for Si atoms within the NPs of the 0.10CaO–0.90SiO$_{2}$ glass model is reported in Table 4 as a function of the NPs’ size. We can firstly observe that outside the NPs, all the silicon atoms are Q$^{4}$, like in a pure amorphous silica melt^50^. Secondly, as the NPs’ size increases, the CaO oxide concentration increases, and the proportion of Q$^{4}$ species decreases to the advantage of Q$^{3}$ and Q$^{2}$ species. The same trend has been previously observed in series of soda-lime-silica glass^51,52^ and in the binary CaO–SiO$_{2}$ system^53^ when the CaO content increases. This trend is consistent with the network modifier role of the Ca$^{2+}$ cation. Moreover, the degree of depolymerization appears to be related to the concentration of calcium oxide in the NPs. An equilibrium seems to be reached for the NPs including 28 Ca atoms and more, which can be explained by the establishment of a short-range order.

**Table 4:** Q$^{\hbox {n}}$ distribution of silicium atoms in the 0.10CaO–0.90SiO$_{2}$ glass structure.

NP size (number of Ca atoms)	Q$^{\hbox {n}}$ [%]				
	Q$^{0}$	Q$^{1}$	Q$^{2}$	Q$^{3}$	Q$^{4}$
0: no Ca	0.00	0.00	0.00	0.01	99.99
1: isolated Ca	0.00	0.04	0.66	22.33	76.97
2–27	0.02	0.34	4.04	32.33	63.27
28–65	0.20	2.07	9.22	35.20	53.31
66–125	0.24	1.98	9.71	34.85	53.22
126+	0.25	2.41	11.21	35.49	50.64

Standard deviations are less than 5%.

**Figure 5:**
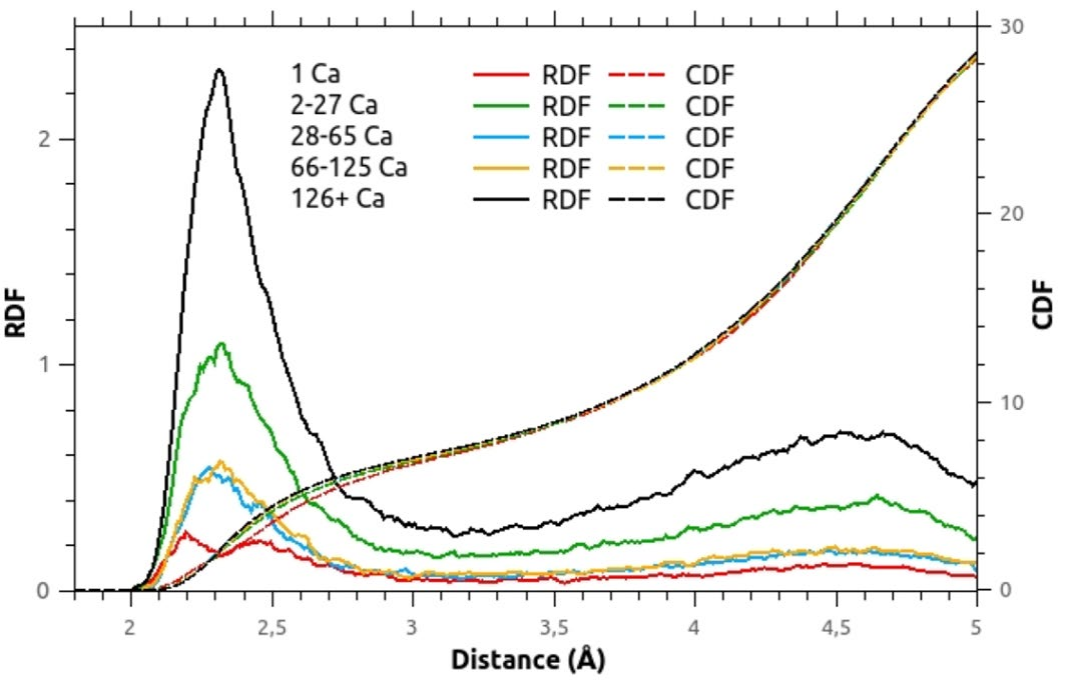
Ca–O RDF and CDF related to the NP size (number of Ca atoms) in the 0.10CaO–0.90SiO$_{2}$ glass model.

In the following part, we discuss the depolymerization of the silica network in the 0.10CaO–0.90SiO$_{2}$ glass model, and more particularly the role of the calcium local environment. Figure 5 displays the Ca–O RDF as a function of the NPs’ size in which Ca atoms are involved. Considering the first peak, we can note that there is no significant difference for NPs including 2 Ca atoms and more: the average Ca–O nearest neighbor distance is 2.34 Å and Ca atoms are surrounded by 7 neighboring oxygen atoms on average. But a drastic change in the RDF shape is observed for the Ca atoms isolated in the silica matrix. The Ca–O RDF is the sum of two main contributions: the first peak is centered around 2.21 Å, corresponding to a majority of NBO* atoms and the second, around 2.45 Å, which is due to the presence of BO* atoms (associated to the second peak). BO* atoms are oxygen atoms bounded to two silicon atoms, which may be the farther from Ca compared to NBO* atoms (oxygen atoms bounded to only one Si atom). This assumption is verified on the Fig. 2.

For a better understanding of the structure of calcium environment, the discrimination of the different oxygen species is applied according to the notation described by Afify et al.^44^. Table 5 reports the oxygen coordination numbers of Ca atoms as a function of the NP’s size in which they are located. The contribution of each oxygen species is displayed, as well as the proportion of N-coordinated Ca sites involved. Oxygen coordination number N$$_{\hbox {Ca}-\hbox {O}}$$ is calculated by counting the number of oxygen atoms within a cutoff distance of 3.05 Å, that corresponds to the first minimum of the Ca–O RDF. One can firstly denote that the first coordination shell of Ca atoms in this glass model is on average composed of 6.90 O. Compared to a Mg codoping of the same concentration^9^, this coordination number is higher ($$\hbox {N}_{\hbox {Mg}-\hbox {O}} = 6.0$$), which is in agreement with the more ionic nature of the Ca–O link, as explained by Hudon & Baker^16^. However, this coordination number depends on the size of the NP: it increases from 6.63 for isolated Ca atoms to 7.02 for Ca atoms included in the largest NPs. The distribution of coordinated sites of calcium atoms is also reported on Table 5. Similarly, with the increase in the NPs’ size, the proportion of 5- and 6-coordinated sites decreases, the 7-coordinated sites become even more predominant, and some 8-coordinated sites appear. One can secondly denote that there is no significant variation for NPs composed of 28 Ca atoms and more, considering the oxygen coordination as well as the different coordinated sites occupied by the Ca$$^{2+}$$ cation. In our modeled structure, we can observe that the oxygen-rich environment found in the NPs is mostly due to the presence of NBO**: with the increase of the NPs’ size, the coordination of Ca with NBO** is favored to the detriment of BO* and NBO*. Besides, in view of the very low amount of NNO atoms found in the NPs, Ca$$^{2+}$$ cation is clearly not able to form its own network, which is in agreement with its network modifier role and explains the mixed composition of the NPs. Since differences between the close environment of Ca and Mg atoms have been revealed in such glass models, the next part is devoted to the comparison of these two network modifier cations in the formation of NPs. In our modeled structure, we can remark that the oxygen-rich environment is mostly due to the presence of NBO$$^{**}$$: with an increase of the NPs size, the coordination of Ca with NBO$$^{**}$$ intensifies to the detriment of BO$$^{*}$$ and NBO$$^{*}$$. The difference in behavior in silica melts between calcium and magnesium will be analyzed and discussed subsequently.

**Table 5:** Interatomic distance Ca–O denoted r (Å), average oxygen’s coordination number of calcium denoted N$_{\hbox {Ca-}}$ and its distribution in the 0.10CaO–0.90SiO$_{2}$ glass model related to the size of the nanoparticle (cutoff Ca–O = 3.05 Å).

NP size (number of Ca atoms)	r (Å)	N$_{\hbox {Ca-}}$					N-coordinated sites (%)					
		${}_{\mathrm{O}}$	${}_{\mathrm{NNO}}$	${}_{\mathrm{NBO*}}$	${}_{\mathrm{NBO**}}$	${}_{\mathrm{BO*}}$	${}_{\mathrm{4}}$	${}_{\mathrm{5}}$	${}_{\mathrm{6}}$	${}_{\mathrm{7}}$	${}_{\mathrm{8}}$	${}_{\mathrm{9}}$
1 *(6.8 %)*	2.21/2.45	6.63	0.00	1.69	0.00	4.94	0.6	6.8	39.8	34.7	18.2	0.0
2-27 *(24.0 %)*	2.34	6.80	0.03	0.63	2.85	3.29	0.0	6.6	31.5	40.7	18.4	2.7
28-65 *(11.6 %)*	2.33	6.98	0.03	0.41	3.82	2.72	0.2	3.8	24.4	45.7	21.2	4.7
66-125 *(12.2 %)*	2.33	7.00	0.04	0.39	3.85	2.72	0.2	3.6	23.2	45.2	24.8	3.0
126+ *(45.4 %)*	2.34	7.02	0.04	0.36	4.00	2.62	0.2	3.3	24.2	43.3	24.3	4.7
All	2.34	6.90	0.04	0.49	3.41	2.96	0.3	4.6	26.6	43.0	21.9	3.7

% of Ca atoms involved in each NP size class are reported in italic text. Standard deviations are less than 5%.

### Comparison between modifiers cations

The structural role of cation in the glass network was classified according to the network hypothesis proposed by Zachariasen^38^ and the Dietzel’s extension, based on field strength values^40^ (FS = Z/a$^{2}$, where Z is the valence of the cation and a = r$_{\hbox {c}}$ + r$_{\hbox {a}}$). According to Dietzel, network modifiers are defined as cations having a low field strength value (FS < 0.4 Å$^{-2}$); intermediates are those with 0.4 $\le$ FS $\le$ 1.0 Å$^{-2}$, while network formers have a field strength higher than 1.5 Å$^{-2}$. Following this classification, calcium (FS = 0.35) has a clear network modifier role, while magnesium (FS = 0.44) has an intermediate role, when both are incorporated in the SiO$_{2}$ matrix. However, based on many studies, Hudon & Baker^16^ affirm that Mg element is not able to copolymerize the silica content, due to its low ionic potential, and consequently do not act as a network former. From these considerations, Ca$^{2+}$ and Mg$^{2+}$ cations should behave similarly in a silica melt. Therefore, comparing our modeled structures including one or the other of these two cations can shed light on this assumption. Results of the analysis of the 0.10MgO–0.90SiO$_{2}$ glass system used in this part can be found from our previous study^9^.

**Figure 6:**
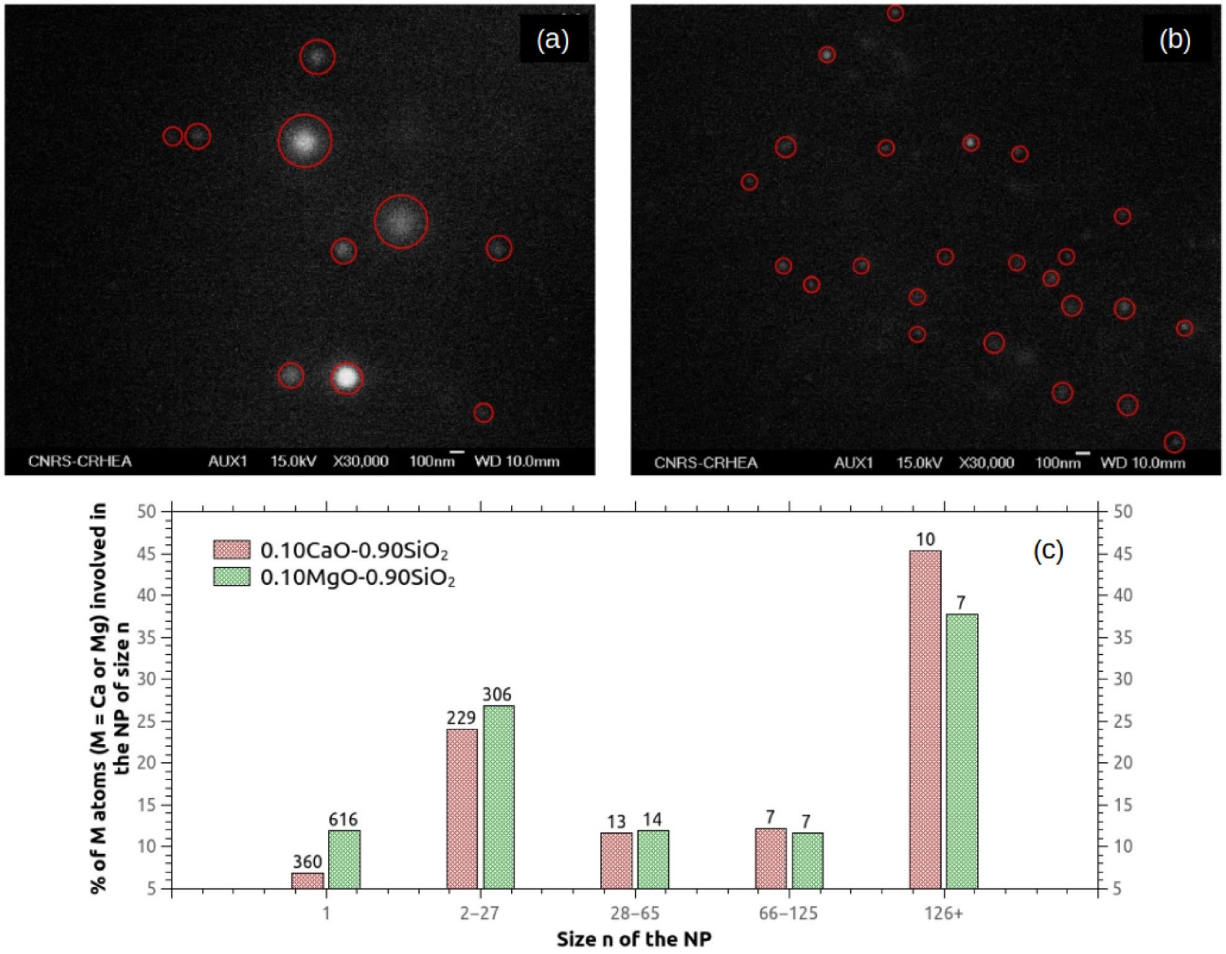
SEM cross section pictures of nanoparticles inside the core of: (**a**) a Ca-codoped silica glass fiber, (**b**) a Mg-codoped silica glass fiber. Red circles indicate the nanoparticle’s location. Glass fibers was elaborated with the same initial conditions and oxide concentration. (**c**) Size distribution of NPs within the modeled 0.10MO–0.90SiO$_{2}$ (M = Ca or Mg) glasses. Number of NPs are indicated as label for each bins.

We firstly propose to compare the size of the NPs in situ formed in the silica matrix with Ca or Mg cations, both from experiments and MD simulations. Table 6 gives the calculated NPs’ volume as a function of their size in the 0.10MO–0.90SiO$_{2}$ (M = Ca or Mg) modeled structures. These volumes are determined using the “Alpha-shape method” algorithm^54^ included in the OVITO program^36^. Experimentally, two glass fibers with the same oxide concentration are elaborated, starting from the same initial condition^12^. Scanning electron microscopy (SEM) method is applied to produce pictures of the NPs embedded in the core of each of the glass fiber, which are displayed in Fig. 6. It is important to note that the transverse section views do not allow to discuss on the potential elongation of NPs along the drawing direction as reported for Mg-based particles^55^. However, a larger diameter section is necessary the consequence of a larger NP. From the modeled structures data of Table 6, Ca-rich nanoparticles appears to be larger than Mg-rich nanoparticles: one can observe that the mean volumes by size class of Ca-containing NPs are 3.1 nm$^{3}$, 6.9 nm$^{3}$ and 19.9 nm$^{3}$, while for Mg-containing NPs, the mean volumes by size class are lower (2.5 nm$^{3}$, 5.3 nm$^{3}$ and 14.0 nm$^{3}$).

**Table 6:** Nanoparticle’s volume interval as a function of their size in the 0.10CaO–0.90SiO$_{2}$ and 0.10MgO–0.90SiO$_{2}$ glass models.

NP size (number of Ca/Mg atoms)	Volume [nm$^{3}$]	
	0.10CaO–0.90SiO$_{2}$	0.10MgO–0.90SiO$_{2}$
28–65	2.1–4.2 (3.1)	1.8–3.2 (2.5)
66–125	4.3–8.2 (6.9)	4.3–7.2 (5.3)
126+	10.6–41.0 (19.9)	7.5–19.8 (14.0)

Mean volume of nanoparticles by size class are represented in parenthesis.

Experimentally, the SEM pictures (Fig. 6) show that Mg-containing NPs are more numerous (as also observed from simulation) but smaller than Ca-containing NPs. Thus, from simulations and experiments, the same trend is observed: Ca-rich silicate NPs appear to be larger than Mg-rich silicate NPs when they are formed in situ through phase separation mechanism in silica melt. A possible explanation for this difference can be attributed to the size of the cation and to the M-O interatomic distance: the Ca$^{2+}$ ionic radius of 1.06 Å (VII)^56^ and the Ca–O mean distance of 2.34 Å, are larger than for Mg$^{2+}$ ($\hbox {r}_{Mg^{2+}}$ = 0.72 Å (VI)^56^, r$_{\hbox{Mg}-\hbox {O}}$ = 2.04 Å$^{3}$).

**Figure 7:**
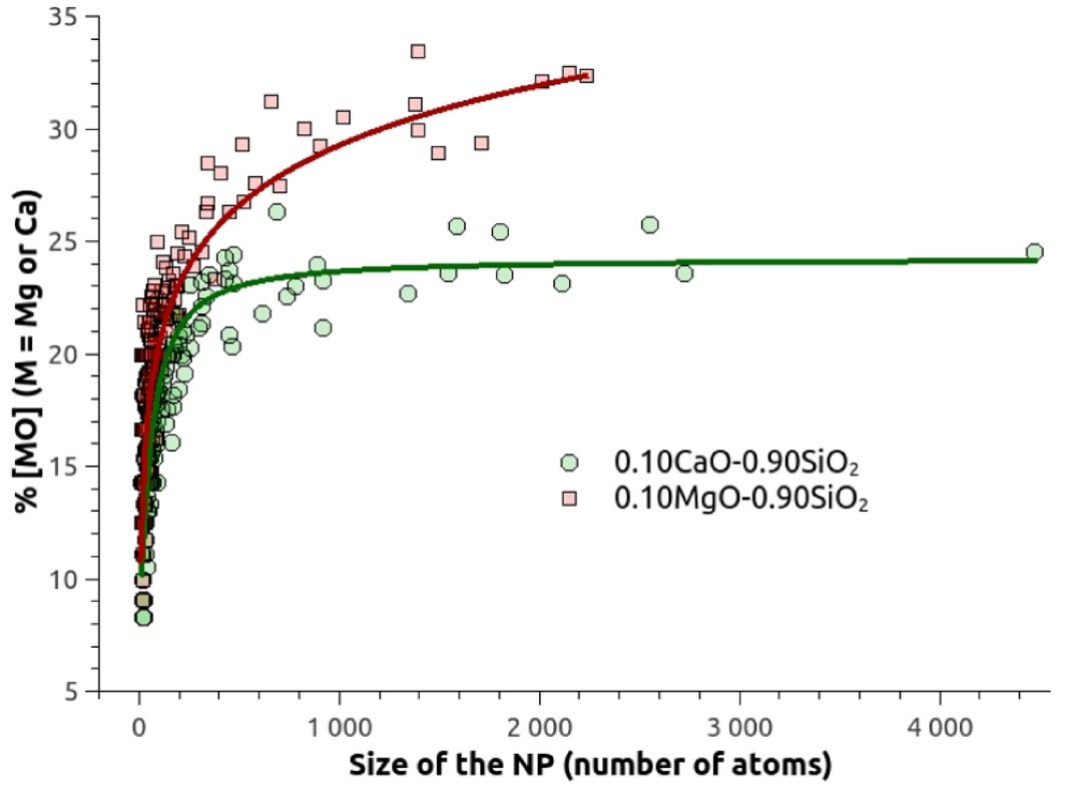
Evolution of the NPs oxide concentration related to the size of the nanoparticle in the 0.10MO–0.90SiO$_{2}$ glass models (M = Ca or Mg).

To have an insight of the link between the NPs’ size and their composition, Fig. 7 represents the evolution of the NPs’ oxide concentration as a function of their size for the two modeled glasses. A clear difference is observed: for the Ca-containing NPs, a concentration plateau of 24 mol% CaO is quickly reached, and no significant variation is observed for NPs larger than 1000 atoms; for the Mg-containing NPs, the MgO concentration keeps growing with the increase of the NPs’ size, and a concentration plateau of $\sim$35 mol% MgO would be reached. Based on phase diagram of SiO$_{2}$–MgO and SiO$_{2}$–CaO binary systems, both concentration plateaus are in accordance with glasses heated at 1775 $^{\circ }$C^57^.

**Figure 8:**
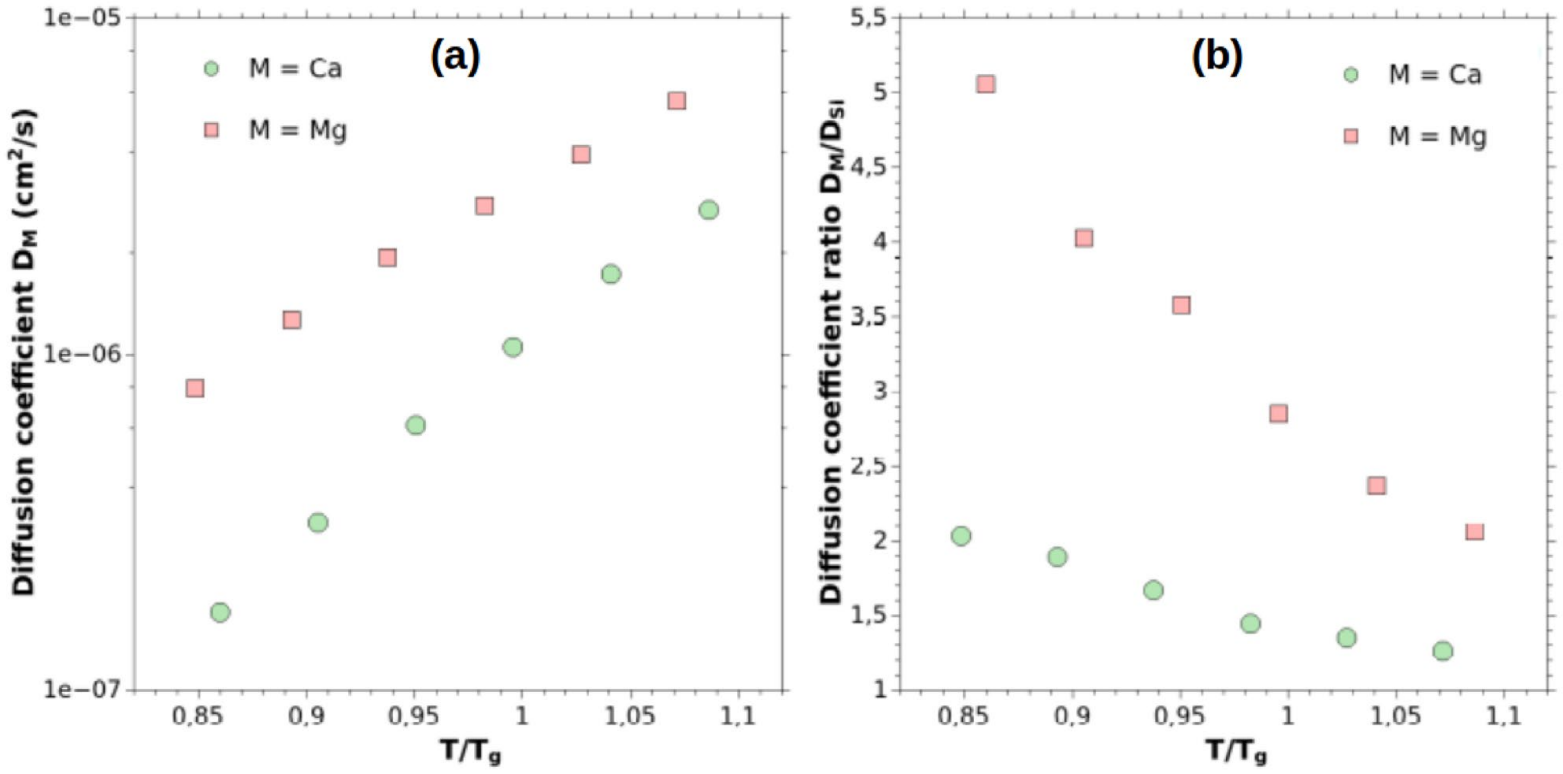
(**a**) Dependence of the diffusion coefficient of the M cation as a function of the temperature, during the phase separation process (between 1900 K and 2400 K). (**b**) Diffusion coefficients ratio of cations in the 0.10MO–0.90SiO$_{2}$ glass model.

To understand the difference in composition variation versus size, the Generalized Gibbs’ approach (GGA) developed by Schmelzer et al.^58–60^ is considered. It describes the new-phase formation processes such as, for example, the mechanism of nucleation-growth in multiple systems. The GGA indicates that at a given temperature, the concentration variation depends on the relative diffusion of the elements. However, this theory does not allow to know the final composition of the NP, nor to say that a more diffusive element will lead to a more concentrated particle. The GGA deals only with the speed with which the “macroscopic” state is reached during the phase transition process. Thus, the lower the diffusion coefficient is, the faster the change of composition will be. To check the consistency of this approach in our context, we have computed and plotted in Fig. 8a the diffusion coefficients of Ca and Mg elements as a function of the temperature, in the range where the phase separation occurs. Here, the glass undergoes a cooling, so the diffusion is calculated in a range of appropriate temperatures. In this range, whatever the temperature, we observe that Mg cations are more diffusive than Ca cations in the silica network, this difference becoming more important at low temperatures. To better understand, the ratio of the diffusion coefficients D$_{\hbox {M}}$/D$_{\hbox {Si}}$ (M = Ca or Mg) as a function of the temperature is displayed in Fig. 8b. Considering the GGA theory, and especially in the case where D$_{\hbox {M}}$ $\gg$ D$_{\hbox {Si}}$, the larger this ratio is, the slower the change in composition will be. Even if we are not in the context of an isothermal treatment, the ratio D$_{\hbox {M}}$/D$_{\hbox {Si}}$ is always higher for Mg compared to Ca. These results imply a slower variation of the composition for Mg-rich NPs compared to Ca-rich NPs.

These results show that, like with Mg, the introduction of Ca into the silica makes it possible to form particles by phase separation following heat treatments. Considering light scattering, the variation in composition as a function of particle size brings back into question the use of the Rayleigh model which only considers a single refractive index for the particles, independently of their sizes. A more general model including a variation of the refractive index as a function of particle size should therefore be developed to account for the optical properties of these systems. Light scattering is based on the characteristics of the particles, in particular their sizes and their refractive indices. The results reported in this article show that the introduction of calcium should induce greater light scattering than magnesium. Indeed, at an identical concentration of alkaline earth ions, doping with calcium makes it possible to obtain larger particles than with doping with Mg. Moreover, in a silica glass, the refractive index increases linearly with the concentration of MgO and CaO^61,62^. Considering plateau values of concentrations in the nanoparticles of 35 mol % MgO and 25 mol % CaO, the expected index increase is 0.07 and 0.09, respectively. Although the maximum concentration is lower in Ca, its higher polarizability contributes to a greater increase in the refractive index. From the luminescence point of view, studies are underway to assess the changes in the environment of rare earth ions in the CaO–SiO$_{2}$ system and to determine whether the lower change in composition compared to Mg is detrimental for the properties of luminescence.

## Conclusions

From MD simulations, Ca-containing nanoparticles are produced in situ in a silica glass (0.10CaO–0.90SiO$_{2}$) through a phase separation mechanism. Investigations on the modeled structures reveal the presence of inhomogeneously distributed amorphous NPs with a wide range of sizes, as observed from experiments^10,63^, and MD simulations^9^ of MgO codoped silica matrix. A detailed analysis of the NPs indicates that they are made of NBO-rich environments where Ca$^{2+}$ cations are surrounded by on average 7 oxygen atoms. Besides, it is shown that Ca-containing NPs are larger than Mg-containing NPs in silica glass. Another important feature is the size-dependent chemical composition of these NPs. However, this size-dependence is less important for Ca-containing NPs than for Mg-containing ones. This difference in behavior can be explained thanks to the GGA theory of Schmelzer et al.^58–60^, also allowing to justify the highest oxide concentration encountered in the Mg-containing NPs. All these features, drawn from MD simulations, make this simple transferable potential a relevant tool for the development of new applications based on nanoparticle-based fibers. For instance, a compromise between size and composition of nanoparticles are required for incorporation of rare-earth ions in order to design new amplified optical fiber devices (where nanoparticles need to be the smallest as possible to minimize light scattering but with a high modification of the composition to alter luminescence properties) or in the development of numerous applications such as sensing systems based on enhanced scattering silica fiber.

## Supplementary Information

Supplementary Information.

## Data Availability

The datasets generated and analysed during the current study available from the corresponding author on reasonable request.
