# Supplementary material for: Phase-separated Ca and Mg-based nanoparticles in SiO2 glass investigated by molecular dynamics simulations
Source: Sci Rep. 2022 Jul 13;12:11959. doi: 10.1038/s41598-022-16139-w (PMC9279444; doi:10.1038/s41598-022-16139-w)
Supplement: Supplementary file 1 — Supplementary Information. [file 41598_2022_16139_MOESM1_ESM.pdf]

Supplementary Information:

Phase-separated Ca and Mg-based nanoparticles in SiO_2 glass investigated by Molecular Dynamics simulations.

Jorel Fourmont[1], Wilfried Blanc[2], Dominique Guichaoua[1], and Stéphane Chaussedent[1,*]

[1]Univ Angers, LPHIA, SFR MATRIX, F-49000 Angers, France

[2]Université Côte d'Azur, CNRS, Institut de Physique de Nice (INPHYNI), UMR 7010, 06108 Nice, France

*stephane.chaussedent@univ-angers.fr

New force-field for phase separation.

From the paper defining the adaptative version of the Pedone potential[S1], Figure 1 displays snapshots of glasses with $0.10MgO\text{-}0.90SiO_2$ chemical composition for a total of 37584 atoms (1296 Mg atoms) and a volume of approximately 100×50×100 $Å^3$. In order to highlight how the adaptative version contributes to phase separation, two glasses have been modelled in the same conditions (melt/quench from 4000 K to 300 K, quenching rate: 5×10^{11} K/s) using (a) Pedone potential[S2] and (b) the adaptative version. To ensure that it is not a matter of the quench that would be too short, a very long simulation with many temperature stages as long as 6.4 ns each (within the phase separation temperature window) is performed using the Pedone potential (c) and its adaptative version (d). More statistical details can be found in reference S1.

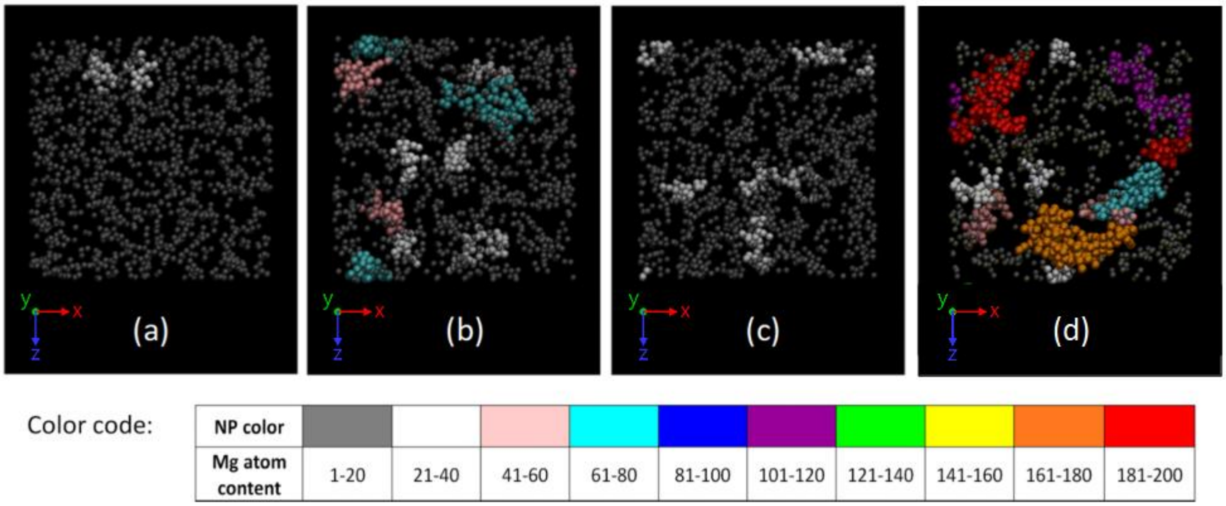

Figure 1: Snapshots of $0.10MgO\text{-}0.90SiO_2$ glasses extracted and adapted from Bidault *et al.*[S1]. (a) Fixed-charge model from Pedone[S2], (b) adaptative potential used in this study, (c) fixed-charge model from Pedone[S2] and (d) adaptative potential with many temperature stages as long as 6.4 ns each (within the phase separation temperature window). Only Mg atoms are shown, but a color code is applied to discriminate them according to the size of the NP in which they are located.

Experimental fabrication of glasses.

Preforms were fabricated by the conventional MVCD technique. In this process, gaseous chlorides ($SiCl_4$, $GeCl_4$, $POCl_3$) are passed through a rotating silica tube, heated by an external burner in translation along the tube. Due to the high temperature, chlorides oxidize, forming particles which deposit on the inner wall. This porous layer turns into a glassy layer when the burner passes over it (the tube external temperature is around 1500 °C). In the final stage, the tube is collapsed into a rod at a temperature higher than 1800°C. Fibres were obtained by stretching preforms in a drawing tower at temperatures higher than 2000 °C under otherwise normal conditions. Opto-geometric properties of the perform (or fibre) are determined by adjusting the composition and number of layers. In our samples, phosphorous and germanium concentrations are ~1 mol% and 2 mol%, respectively.

Erbium and alkaline-earth ions were incorporated through the solution doping technique. An alcoholic solution (of desired strength of $ErCl_3.6H_2O$ and $MCl_2.6H_2O$, M=Ca or Mg) is soaked for two hours in the unsintered core layer. After removing the solution, the layer is dried and sintered. Erbium concentration is estimated through absorption spectra to be around 100 ppm. Alkaline-earth ions concentration in the solution was 0.1mol/l for both preforms. The concentration has been measured by Electron Probe Micro Analysis (EPMA) in the fibers. It is about 0.15mol % MO (M = Ca or Mg). Alkaline-earth ions concentration is much higher in the MD simulations than in the samples to ensure a statistical relevance of the simulated results.

References.

(S1) Bidault, X., Chaussedent, S. & Blanc, W. A simple transferable adaptive potential to study phase separation in large-scale xMgO-(1-x)SiO2 binary glasses. The J. Chem. Phys. 143, 154501, DOI: https://doi.org/10.1063/1.4932984 (2015).

(S2) Pedone, A., Malavasi, G., Menziani, M. C., Cormack, A. N. & Segre, U. A new self-consistent empirical interatomic potential model for oxides, silicates, and silica-based glasses. The J. Phys. Chem. B 110, 11780–11795, DOI: https://doi.org/10.1021/jp0611018 (2006).
